# Swine influenza A replicon particle and live attenuated influenza virus vaccines induce differential systemic and mucosal antibody and T cell responses

**DOI:** 10.3389/fvets.2025.1690418

**Published:** 2026-01-30

**Authors:** Meghan Wymore Brand, Bryan S. Kaplan, Carine K. Souza, Jayne E. Wiarda, J. Brian Kimble, Bailey Arruda, Daniel R. Perez, Amy L. Baker

**Affiliations:** 1 Virus and Prion Research Unit, National Animal Disease Center, USDA-ARS, Ames, IA, United States; 2 Department of Population Health, Poultry Diagnostic and Research Center, University of Georgia, Athens, GA, United States

**Keywords:** antibody, influenza A, mucosal, swine, T cells, vaccines

## Abstract

Influenza A virus (IAV) in swine is a significant economic concern, and there is a critical need to improve vaccine efficacy. Commercial and experimental vaccine platforms are effective against homologous infection but may not reliably provide protection against drifted or heterologous viruses. Live attenuated influenza A virus (LAIV) vaccines induce mucosal antibody and localized cellular immune responses that may provide partial protection from drifted IAV. However, limited data exist on the induction of mucosal antibody and cellular immune responses and heterologous protection induced by RNA-based vaccines in swine. In this work, experimental, non-adjuvanted hemagglutinin-based replicon particle (RP-HA), and live attenuated influenza A virus (LAIV) vaccines were assessed for induction of mucosal antibody, cellular immune responses, and heterologous protection. LAIV reduced viral shedding and viral lung load while RP-HA limited macroscopic lung lesions. Both vaccines induced similar homologous systemic antibody and mucosal IgG, while only LAIV induced high levels of mucosal IgA. Both vaccines stimulated *ex vivo* virus-specific T cell proinflammatory cytokine production and proliferation. LAIV induced greater CD8^+^ T cell responses in the blood and the lungs, and CD4^+^ T cells in the blood, though RP-HA induced higher lung CD4^+^ T cell cytokine responses. Together, these results demonstrate that LAIV and RP-HA IAV vaccines induce differential antibody and T cell responses that are likely impacted by vaccine platform and route of exposure. A better understanding of correlates of protection, such as cellular immunity and mucosal antibody induction, will aid in the development of improved swine IAV vaccination strategies.

## 1 Introduction

Influenza A (IAV) is an endemic swine respiratory pathogen with a significant economic impact on the swine industry. H1N1, H1N2, and H3N2 IAV subtypes are endemic in U.S. swine populations and diversified into divergent genetic clades over time (1, 2). Thus, there is a critical need to better understand vaccine correlates of protection to improve vaccination strategies, broadening IAV immunity against multiple clades and subtypes. IAV has two major surface glycoproteins, hemagglutinin (HA) and neuraminidase (NA), which are the main viral targets for induction of antibody responses. Serum antibody to HA, measured by hemagglutinin inhibition assay, has been established as the gold standard correlate of protection for influenza vaccines. Traditional adjuvanted inactivated swine vaccines rely on the induction of high levels of systemic neutralizing antibody, which is highly effective in providing protection from homologous viral infection, though cross-reactivity to drifted or heterologous HA varies (1, 3). Recently, the role of antibody to NA has begun to be explored in vaccine protection and may provide partial protection even with a heterologous HA (4–7).

Swine IAV vaccine immunity is dependent on the platform and route of exposure (3). Adjuvanted IAV whole inactivated virus (WIV) vaccines are heavily used in the U.S. swine industry, in addition to replicon particle (RP) HA- and NA-based vaccines (3, 7–9). IAV WIV vaccines induce protective homologous antibody levels but limited protection against heterologous IAV, presumably due to the absence of T cell responses and neutralizing antibodies in the mucosa (3, 10). Further, IAV vaccine-associated enhanced respiratory disease (VAERD) may be induced with a mismatched WIV due to the lack of matched neutralizing antibody responses and cytokine dysregulation that leads to immunopathology in the lungs (10–13). Multiple live attenuated influenza virus (LAIV) platforms have been experimentally evaluated in swine that are highly effective against homologous virus challenge and provide partial protection from heterologous viruses without induction of VAERD (3, 14–19). However, a commercially licensed LAIV has been demonstrated to reassort with wild-type viruses in the field (3, 20). HA- and NA-based RP vaccines represent a newer IAV vaccine technology platform for U.S. swine that can be rapidly customized and produced specifically to the strains circulating on the farm. RP vaccines induce robust HA and NA antibodies without causing VAERD with heterologous infection (6, 7, 21–24).

Cellular immune responses are known to be important in shaping immunity to IAV. Previous assessments of cellular immune responses in swine IAV vaccine protection are primarily for LAIV, with limited evaluation in heterologous infections (10, 25–28). A few studies demonstrate induction of T cell responses for RP vaccines in swine (7, 22, 29). Multiple research studies demonstrate swine CD4^+^ and CD8^+^ T cells are activated after infection with IAV and are likely integral in vaccine protection, especially in heterologous infection (25, 30–34). Additionally, the potential role of mucosal antibody as a correlate of protection is poorly defined for swine IAV vaccines (32, 35). Many vaccine studies have demonstrated that experimental LAIV vaccines generate mucosal antibody in addition to homologous serum antibody, though there is limited data on mucosal antibody induced by swine RP vaccines (7, 10, 15–18, 22, 26, 28, 29, 36). Thus, we evaluated swine LAIV and RP-HA in protection from antigenically divergent IAV challenge and induction of responses and mucosal antibody. Though there was a limited number of animals in the study, this study still demonstrated that swine LAIV and RP-HA IAV vaccines induced differential antibody and T cell responses, and these vaccines had differential impacts on heterologous protection as LAIV limited viral shedding and viral lung load, while RP-HA limited macroscopic lung lesions. Although neutralizing antibody is a strong correlate of protection for homologous viruses, increased CD8^+^ T cell responses in LAIV-vaccinated pigs associated with decreased virus shedding may indicate a correlate of protection for heterologous infection. These data emphasize the importance of understanding swine cellular immune responses as correlates of protection in heterologous IAV vaccine immunity.

## 2 Materials and methods

### 2.1 Viruses and vaccines

A/California/04/2009 H1N1 clade 1A.3.3.2 (CA/09) and A/swine/Minnesota/02011/2008 H1N2 clade 1B.2.2 (MN/08) were the viruses utilized for this study. Representatives from these clades continue to circulate in U.S. swine (37). CA/09 LAIV was previously constructed by using reverse genetics to insert the CA/09 HA and NA into the temperature-sensitive, attenuated backbone of A/turkey/Ohio/313053/04 H3N2 (14, 15). The LAIV vaccine was propagated as previously described in MDCK cells (16). Non-adjuvanted, replicon particle vaccine containing CA/09 HA was provided by Merck Animal Health (Ames, IA, United States) (38). This RNA particle, alphavirus-based vaccine platform (marketed as Sequivity^®^) has been reviewed elsewhere (22, 24, 38–40). The RP vaccine was stored at −80 °C and thawed overnight at 4 °C on ice. Both vaccines were maintained on ice until use. MN/08 and CA/09 viruses for challenge and immune assays were propagated in MDCK cells in OptiMEM (Gibco, Thermo Scientific, Waltham, MA) supplemented with antibiotic-antimycotic (Thermo Scientific, Waltham, MA) and 1 μg/mL L-1-Tosylamide-2-phenylethyl chloromethyl ketone (TPCK, Worthington Biochemicals, Lakewood, NJ). A no-virus mock culture of OptiMEM on MDCK cells was also prepared for cell stimulations.

### 2.2 Animal study design

Mixed-sex 3-week-old piglets were obtained from a herd free of porcine reproductive and respiratory syndrome virus (PRRSV) and IAV. On arrival, the piglets were treated with ceftiofur crystalline free acid (Zoetis, Parsippany, NJ) and tulathromycin (Zoetis, Parsippany, NJ), and were confirmed influenza seronegative with an IAV nucleoprotein ELISA kit (Swine Influenza Virus Ab Test, IDEXX, Westbrook, ME). Animals were housed in biosafety level 2 (BSL2) containment and cared for in compliance with the National Animal Disease Center’s Institutional Animal Care and Use Committee.

The pigs were randomly assigned to treatment groups (*n* = 9 or 10), and received either RP-HA vaccine, LAIV vaccine, or no vaccine (Table 1). Table 2 depicts the sample collection timeline. The first vaccine dose was given at 4 weeks of age and then boosted at 7 weeks of age. RP-HA was administered intramuscularly in the neck with an 18-gauge needle, 1 × 10^7^ RP-HA in a 1-mL dose. LAIV vaccine was administered with a MAD nasal intranasal mucosal atomization device (Teleflex, Morrisville, NC), 1 × 10^6^ 50% tissue culture infective dose (TCID_50_) per ml, 1 mL per nostril. At 10 weeks of age (3 weeks post-boost), pigs were sedated with an intramuscular injection of a cocktail of ketamine (8 mg/kg of body weight; Phoenix, St. Joseph, MO), xylazine (4 mg/kg; Bayer, Whippany, NJ), and telazol (6 mg/kg; Zoetis, Parsippany, NJ), and challenged with MN/08 virus, 2 mL intratracheally and 1 mL intranasally at 1×10^5^ TCID_50_/mL (41). Nasal swabs (NS; FLOQSwabs, Copan, Murrieta, CA) were collected at 0, 3, and 5 days post-infection (dpi), and serum at −2 dpi (pre-challenge) (41). Five pigs from each group were humanely euthanized with a lethal dose of pentobarbital (Fatal Plus, Vortech Pharmaceuticals, Dearborn, MI) at 5 dpi. The remaining pigs were maintained for other purposes. At necropsy, the lung tissue was excised, percentages of macroscopic lesions were estimated, and bronchoalveolar lavage fluid (BALF) was collected (41). Additionally, the right-middle or most affected lung lobe and tracheal tissues were preserved in 10% neutral-buffered formalin for histologic assessment (41).

**Table 1:** Study design groups.

Group	Vaccine	Challenge
NV/NC	None	None
RP-HA	RP-HA CA/09	MN/08
LAIV	LAIV CA/09	MN/08
NV/C	None	MN/08

**Table 2:** Sample collection timeline.

Day of study	Vaccination	Infection	Serum	Nasal swab	BALF	T cell stimulation	Necropsy and lesion scoring
6 WPI	X						
3 WPI	X						
0 DPI		X	X	X			
1 DPI				X			
3 DPI				X			
5DPI				X	X	X	X

WPI, weeks pre-infection; DPI, days post-infection.

### 2.3 Macroscopic and microscopic lesions

At necropsy, lungs were macroscopically scored for lesions based on the affected lung, weighted to proportions of total lung volume. Briefly, individual lung lobes were evaluated for percent of total lung lobe lesioned tissue, and then percent was weighted based on the approximate total lung volume (Supplementary Table 1), for a composite estimation of lesioned tissue of the total lungs (42). Formalin-fixed lung and tracheal tissues were processed and stained with hematoxylin and eosin, and a veterinary pathologist blinded to treatment groups evaluated tissues for microscopic lesions to generate a composite score of 0–20 (Supplementary Table 2) (43). Tracheal tissues were scored for the presence of epithelial necrosis and tracheitis, for a composite score of 0–8 (Supplementary Table 3) (16).

### 2.4 Virus detection and diagnostic microbiology

Virus detection in nasal swab fluid and BALF was performed on MDCK cells with immunocytochemistry as previously described (12, 44). Briefly, BALF samples and filtered nasal swab fluid were diluted and inoculated onto MDCK monolayers. After 48 h, cells were fixed with 4% phosphate-buffered formalin in PBS and stained with an antibody specific for IAV nucleoprotein (12, 44). Samples positive in the virus isolation were further titrated to determine TCID_50_/mL using the Reed and Muench method (12, 45).

BALF was screened for aerobic bacteria by culture on blood agar and Casmin (NAD-enriched) plates for 48 h at 37 °C to confirm the absence of bacterial respiratory pathogens. Additionally, BALF was screened for additional pathogens with PCR. RNA was extracted with the MagMax CORE Isolation Kit (Thermo Fisher Scientific, Waltham, MA) with a KingFisher-96 Apex (Thermo Fisher Scientific, Waltham, MA). Commercial PCR assays were performed for *Mycoplasma hyopneumoniae* (VetMax *M. hyopneumoniae* reagents, Thermo Fisher Scientific, Waltham, MA) and PRRSV (VetMax NA and EU PRRSV Reagents, Thermo Fisher Scientific, Waltham, MA) with the VetMax-Plus qPCR Master Mix (Thermo Fisher Scientific, Waltham, MA), as per manufacturer’s recommendations on an Applied Biosystems 7,500 Real Time PCR System (Thermo Fisher Scientific, Waltham, MA). qPCR for porcine circovirus 2 and 3 was performed with an assay from the Iowa State University Veterinary Diagnostic Lab (kindly provided by Phil Gauger).

### 2.5 Serologic and mucosal antibody assays

Pre-challenge serum was assessed for HA-, NA-, and whole virus-specific antibodies. For hemagglutinin inhibition (HI) assays, serum was treated with receptor-destroying enzyme II (Hardy Diagnostics, Santa Maria, CA), heat-inactivated at 56 °C, treated with 20% Kaolin (Sigma Aldrich, St. Louis, MO), absorbed with 50% turkey red blood cells (RBCs), and used at 1:10 dilution. HI assay was performed with MN/08 and CA/09 wild-type viruses using 0.5% turkey RBCs, as previously described (46). HI results were reported as geometric mean antibody titers.

NA-specific antibodies were assessed with neuraminidase inhibition by enzyme-linked lectin assay (ELLA). H9N1 or H9N2 viruses were reverse engineered (rg) with an HA gene from A/guinea fowl/HK/WF10/1999 H9N2 (47). The N1 antigen was derived from the NA from A/California/04/2009 pdmH1N1 and engineered on the backbone of A/Puerto Rico/8/1934, and the N2 from A/swine/Minnesota/02011/2008 was engineered on the backbone of the attenuated internal genes from A/turkey/Ohio/313053/2004 H3N2 (48). The NI titer was defined as the geometric mean titer of the highest twofold dilution resulting in 50% inhibition of NA activity (47).

Whole virus-specific IgG and IgA ELISA was performed on pre-challenge serum and 5 dpi BALF for MN/08 and CA/09 wild-type virus in duplicate as previously described, with the modification of 150 μL Starting Block (Thermo Fisher Scientific, Waltham, MA) for blocking for BALF (49, 50). Results were reported as average optical density (O.D.) for duplicate wells.

### 2.6 PBMC isolation

Blood was collected in heparin tubes (BD, Franklin Lakes, NJ) at 5 dpi for peripheral blood mononuclear cells (PBMC) isolation. Blood was diluted 1:1 with room temperature Dulbecco’s Phosphate Buffered Saline (DPBS) with 2% FBS (StemCell Technologies, Vancouver, Canada) and carefully layered on the top of a 50-ml SepMate tube (StemCell Technologies, Vancouver, Canada) prefilled with Lymphoprep (StemCell Technologies, Vancouver, Canada). Tubes were centrifuged at 1200x*g* for 10 min at room temperature. The buffy coat was gently poured off to a new 50-ml tube (Falcon, Corning, Glendale, AZ) and cells were washed twice with DPBS (Gibco, ThermoFisher, Waltham, MA), and residual red blood cells lysed with filter-sterilized, pH 7.2 ACK lysis buffer of 0.15 M ammonium chloride (Sigma-Aldrich, St. Louis, MO), 10 mM potassium bicarbonate (Sigma-Aldrich, St. Louis, MO), and 0.1 mM EDTA (Invitrogen, ThermoFisher, Waltham, MA). Cells were washed again, strained through a 40-μm strainer (Falcon, Corning, Glendale, AZ), and resuspended in 1 mL of Advanced RPMI 1640 (ThermoFisher, Waltham, MA) with 10% heat-inactivated FBS (Seradigm, VWR, Radnor, PA), 1% L-glutamine (Gibco, ThermoFisher, Waltham, MA), 1% Penicillin–Streptomycin (Gibco, ThermoFisher, Waltham, MA), 1% HEPES (Gibco, ThermoFisher, Waltham, MA), 2% essential amino acids (Gibco, ThermoFisher, Waltham, MA), and 0.05 mM 2-beta mercaptoethanol (ThermoFisher, Waltham, MA). Cells were counted with a Countess II (Life Technologies, Carlsbad, CA) per manufacturer’s instructions after a 1:10 dilution.

### 2.7 Lung cell isolation

An approximately 3x3x3 cm piece of right cranial lung lobe adjacent to the tissue taken for histopathology was placed in a 50-ml tube with PBS at necropsy and maintained on ice. The tissue was first cut into smaller pieces with scissors then placed into tissue dissociator tubes (C tubes, Miltenyi Biotec, Auburn, CA) with RPMI 1640 with stable glutamine (ThermoFisher, Waltham, MA), 2% heat-inactivated FBS (Seradigm, VWR, Radnor, PA), 1% Penicillin–Streptomycin (Gibco, ThermoFisher, Waltham, MA), 20 mM HEPES (Gibco, ThermoFisher, Waltham, MA), 25 U/mL DNase type I (Invitrogen, ThermoFisher, Waltham, MA), and 300 U/mL collagenase type 1 (Gibco, ThermoFisher, Waltham, MA). The m_lung_01 protocol was performed on a gentleMACs Octo Dissociator (Miltenyi Biotec, Auburn, CA), then a 30 min incubation shaking at 37 °C, and then the m_lung_02 protocol on the dissociator. The digested tissues were poured through a 70-μm cell strainer (MACS SmartStrainers, Miltenyi Biotec, Auburn, CA) and washed with PBS. Residual red blood cells were lysed, washed, strained, and counted as previously described for PBMCs.

### 2.8 Virus-specific cytokine production

Isolated PBMCs were stimulated in a 96-well round bottom plate, 5×10^5^ cells per well, (Falcon, ThermoFisher, Waltham, MA) with media, mock (uninfected cell culture supernatant), CA/09 (multiplicity of infection [MOI] 0.1), MN/08 (MOI 0.1), or PMA/Ionomycin (Cell Activation Cocktail without Brefeldin A, BioLegend, San Diego, CA) for 18 h at 37 °C CO_2_ 5%. At 18 h of incubation, Brefeldin A was added (Protein Transport Inhibitor Cocktail, eBioscience) for an additional 4 h of stimulation prior to staining. Cells were then stained with eFluor 780 Fixable Viability Dye (ThermoFisher, Waltham, MA) at 1:1000 in PBS per manufacturer’s recommendations. Next, cells were stained sequentially with anti-CD8β antibody (1:40, clone PPT23, BioRad, Hercules, CA), anti-mouse IgG1 BV711 (1:100, clone X56, BD Biosciences, San Diego, CA), and a cocktail of anti-CD4a PE-Cy7 (1:15, clone 74-12-4, BD Biosciences, San Diego, CA) and anti-CD3e PE (clone BB23-8E6-8C8, BD Biosciences, San Diego, CA) at room temperature. Cells were then fixed and permeabilized with the BD Cytofix/Cytoperm kit (BD Biosciences, San Diego, CA) per manufacturer’s instructions, followed by intracellularly staining with a cocktail of anti-IFN-*γ* PerCP-Cy5.5 (1:15, clone P2G10, BD Biosciences, San Diego, CA) and anti-TNF-*α* BUV 395 (1:15, clone MAb11, BD Biosciences, San Diego, CA) on ice. Fluorescence minus one and single-stained samples were performed as staining controls, and anti-mouse IgG1, *κ* BUV395 (BD Biosciences, San Diego, CA) and anti-mouse IgG1, κ PerCp-Cy 5.5 (BD Biosciences, San Diego, CA) were used as isotype controls for cytokine staining. Stained cells were resuspended in PBS with 0.1% Bovine Serum Albumin (Sigma Aldrich, St. Louis, MO) and kept at 4 °C until analysis on a BD FACSymphony A5 flow cytometer (BD Biosciences, San Diego, CA). FlowJo software v 10.9 (Tree Star, Inc., Ashland, OR) was used for data analysis.

### 2.9 Virus-specific proliferation stimulation

Isolated lung cells were stained with CellTrace Violet (Invitrogen, ThermoFisher Scientific, Waltham, MA) as per the manufacturer’s recommendation and plated at 5×10^5^ cells per well in a 96-well round-bottom plate. Cells were stimulated with media, mock no-virus, CA/09 (MOI 0.1), MN/08 (MOI 0.1), or pokeweed mitogen (2 μg/mL, ThermoFisher Scientific, Waltham, MA) for 6 days at 37 °C CO_2_ 5%. Cells were then stained as above with viability dye, anti-CD8β, anti-mouse IgG1, anti-CD4a, and anti-CD3e PE and fixed before flow cytometry. Fluorescence minus one and single-stained samples were performed as staining controls. Data were analyzed with FlowJo Software v 10.9 (Tree Star, Inc., Ashland, OR).

### 2.10 Statistical analysis and data availability

Results were analyzed with Prism 9 (GraphPad, San Diego, CA) with an analysis of variance (ANOVA) test. Variables with significant effects by treatment group were subjected to pairwise mean comparisons using the Tukey–Kramer test. Data associated with this study are available at the USDA Ag Data Commons at https://doi.org/10.15482/USDA.ADC/29591348.

## 3 Results

### 3.1 Experimental RP-HA reduced macroscopic lesion scores and LAIV reduced viral titers after heterologous challenge

**Figure 1:**
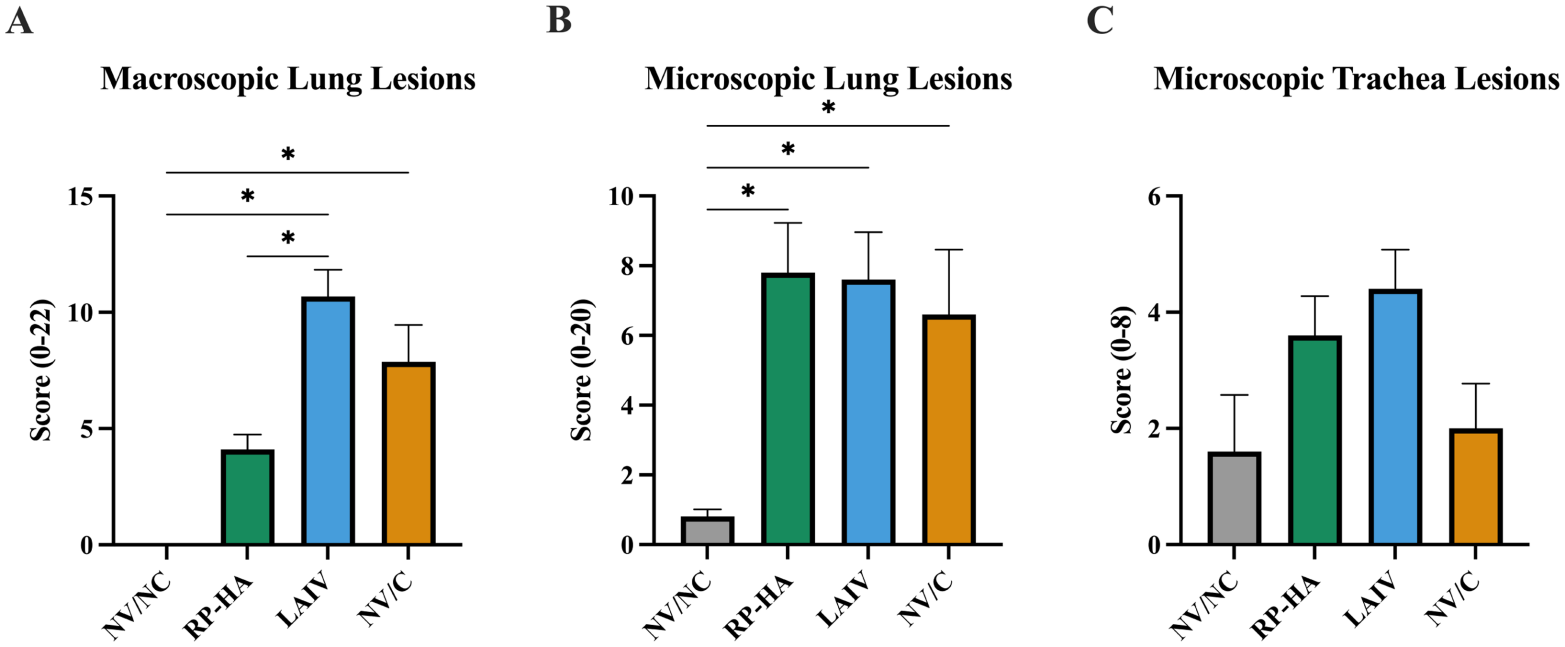
Lesions at 5 days post-infection. Estimated macroscopic lung lesion percentages were visually estimated **(A)**. Microscopic lung **(B)** and trachea **(C)** lesions were evaluated by a veterinary pathologist. NV, non-vaccinated; NC, non-challenged; RP, replicon particle; HA, hemagglutinin; LAIV, live attenuated influenza virus; C, challenged. Data are presented as mean ± standard error of the mean (*n* = 5). Statistically significant differences (*p* ≤ 0.05) between means are indicated by lines and asterisks.

**Figure 2:**
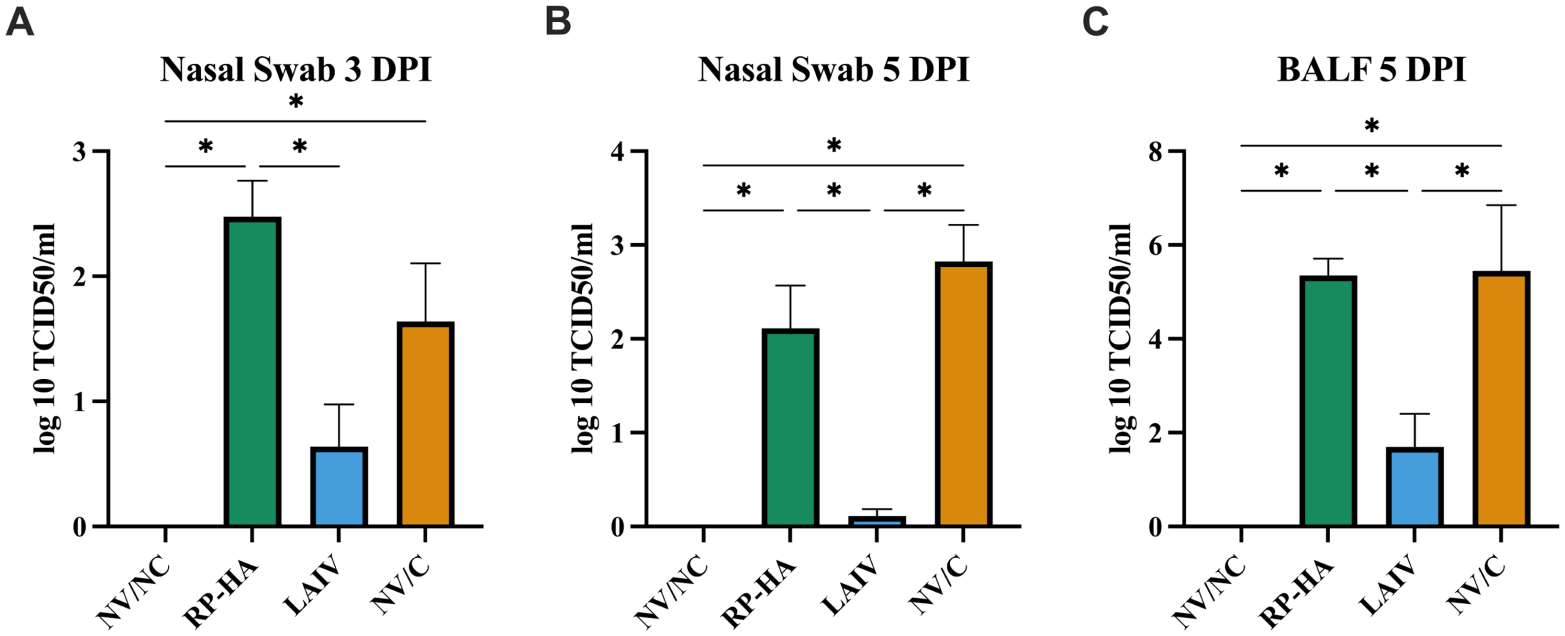
Viral titers in pigs following challenge. Nasal swabs were collected at 3 **(A)** and 5 **(B)** days post-infection. Bronchoalveolar lavage fluid was collected at necropsy **(C)**. NV, non-vaccinated; NC, non-challenged; RP, replicon particle; HA, hemagglutinin; LAIV, live attenuated influenza virus; C, challenged; DPI, days post infection. Data are presented as mean ± standard error of the mean (nasal swabs *n* = 9–10; BALF *n* = 5). Statistically significant differences (*p* ≤ 0.05) between means are indicated by lines and asterisks.

At 5 dpi, macroscopic lesion scores were significantly reduced in RP-HA vaccinated pigs compared to LAIV-vaccinated pigs, though not significantly reduced compared to NV/C (Figure 1A). Microscopic lung and trachea lesion scores were equivalent between all infected groups (Figures 1B,C). Viral titers in 3 dpi nasal swabs of LAIV-vaccinated pigs were significantly reduced compared to RP-HA-vaccinated pigs and had a trend for reduced viral titers compared to NV/C (Figure 2A). LAIV-vaccinated pigs had significantly reduced viral titers in 5 dpi nasal swabs and 5 dpi BALF compared to RP-HA-vaccinated and NV/C pigs (Figures 2B,C). Viral titers in RP-HA-vaccinated pigs were equivalent to NV/C.

### 3.2 Experimental RP-HA and LAIV vaccines induced homologous systemic antibody responses

**Figure 3:**
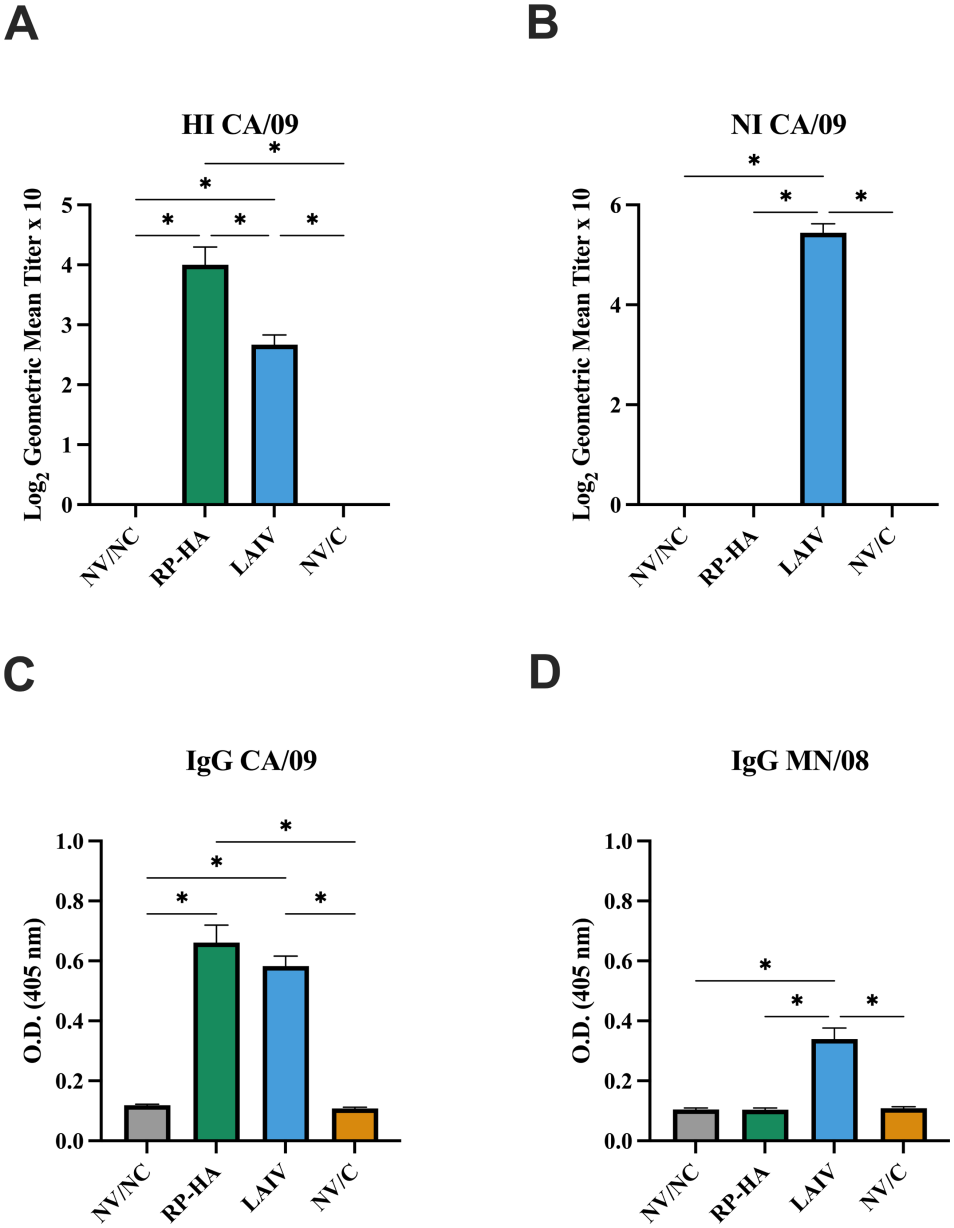
Hemagglutinin, neuraminidase, and whole virus-specific antibody responses in vaccinated pigs prior to infection. Hemagglutinin inhibition titers for CA/09 **(A)** were transformed on log_2_ scale. Neuraminidase inhibition titers to CA/09 **(B)** were assessed by ELLA assay. Optical density (O.D.) of IgG against CA/09 **(C)** and MN/08 **(D)** determined by whole virus ELISA. HI, hemagglutinin inhibition; NI, neuraminidase inhibition; NV, non-vaccinated; NC, non-challenged; RP, replicon particle; HA, hemagglutinin; LAIV, live attenuated influenza virus; C, challenged. Data presented as mean ± standard error of the mean (*N* = 9–10). Statistically significant differences (*p* ≤ 0.05) between means are indicated by lines and asterisks.

Both RP-HA and LAIV vaccines induced homologous HI serum antibody, with significantly higher antibody levels induced by the RP-HA vaccine (Figure 3A). Only the LAIV vaccine induced homologous NI serum antibody (Figure 3B). No cross-reactivity was demonstrated between the CA/09 vaccine and MN/08 challenge virus by HI or NI (data not shown). Both vaccines induced whole virus-specific serum IgG to CA/09, but only LAIV-vaccinated pigs had antibody cross-reactive to MN/08 (Figures 3C,D).

### 3.3 Experimental RP-HA and LAIV vaccines induced differential IgG and IgA in the lung

**Figure 4:**
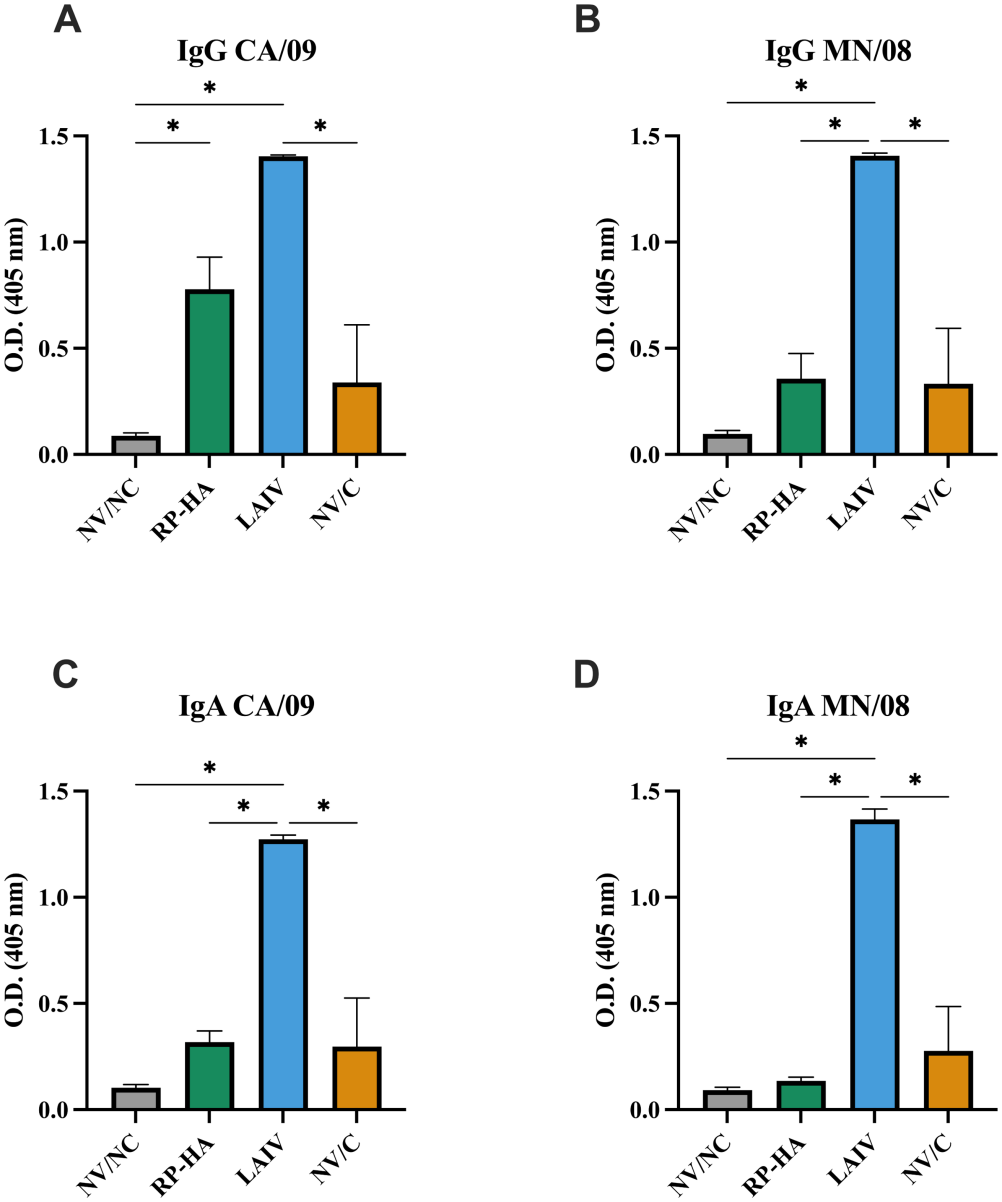
Whole virus-specific antibody in BALF 5 days post-infection. Optical density (O.D.) of IgG **(A,B)** and IgA **(C,D)** against CA/09 and MN/08 determined by whole virus ELISA. NV, non-vaccinated; NC, non-challenged; RP, replicon particle; HA, hemagglutinin; LAIV, live attenuated influenza virus; C, challenged. Data are presented as mean ± standard error of the mean (*N* = 5). Statistically significant differences (*p* ≤ 0.05) between means are indicated by lines and asterisks.

RP-HA- and LAIV-vaccinated pigs had elevated levels of vaccine CA/09 virus-specific IgG in BALF at 5 dpi compared to NV/NC and NV/C (Figure 4A). LAIV-vaccinated pigs had significantly elevated levels of IgG cross-reactive to MN/08 compared to all other groups (Figure 4B). LAIV-vaccinated pigs also had robust levels of vaccine CA/09 virus-specific IgA levels that were cross-reactive to MN/08 (Figures 4C,D).

### 3.4 Experimental RP-HA and LAIV vaccines induced variable PBMC and lung T cell proinflammatory cytokine responses

**Figure 5:**
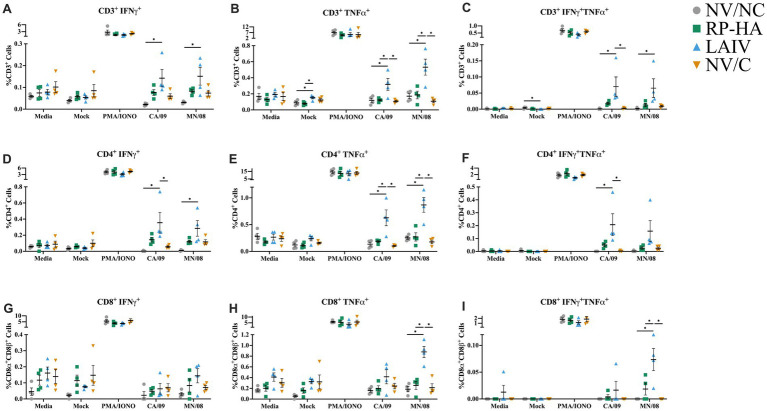
Virus-specific cytokine production of peripheral T cells at 5 days post-infection. Peripheral blood mononuclear cells were stimulated overnight with media, mock, PMA/IONO, CA/09, or MN/08 virus (MOI 0.1). CD3^+^ **(A–C)**, CD4^+^ **(D–F)**, and CD8β ^+^ **(G–I)** production of IFN-*γ* **(A,D,G)**, TNF-*α* **(B,E,H)**, and dual IFN-γ and TNF-α **(C,F,I)**. PMA/IONO, phorbol myristate acetate and ionomycin; NV, non-vaccinated; NC, non-challenged; RP, replicon particle; HA, hemagglutinin; LAIV, live attenuated influenza virus; C, challenged; MOI, multiplicity of infection. Data presented as mean ± standard error of the mean (*N* = 4). Statistically significant differences (*p* ≤ 0.05) between means within each stimulation treatment are indicated by lines and asterisks.

PBMCs were isolated at 5 dpi to correspond to lung cell and mucosal antibody responses post-challenge. PBMCs were stimulated *ex vivo* with wild-type CA/09 vaccine virus or wild-type MN/08 challenge virus. Specific T cell populations (CD3e, CD4a, and CD8β positive cells) were evaluated for production of IFN-*γ* and TNF-*α* with flow cytometry (Figure 5). The gating scheme for this analysis is shown in Supplementary Figure 1. Cytokine production from PBMCs of RP-HA-vaccinated pigs was statistically similar to NV/NC and NV/C, while significant increases were noted for LAIV-vaccinated pigs relative to other groups for several readouts from CA/09 or MN/08 stimulations. Generally, the percentages of cytokine-producing CD3^+^ and CD4^+^ T cells were increased and more variable in LAIV-vaccinated pigs compared to other groups after stimulation with both CA/09 and MN/08 (Figures 5A–F). While CA/09 stimulation did not significantly increase the percentages of cytokine-producing CD8^+^ cells for any treatment groups, CD8^+^ cells from LAIV-vaccinated pigs stimulated with MN/08 had increased TNF-*α* and dual IFN-*γ*/TNF-α production compared to all other groups (Figures 5G–I). As a control for differences in internal genes, PBMCs from LAIV-vaccinated animals were also stimulated with the CA/09 LAIV vaccine, and they were statistically similar to CA/09 stimulation (Supplementary Figure 2).

**Figure 6:**
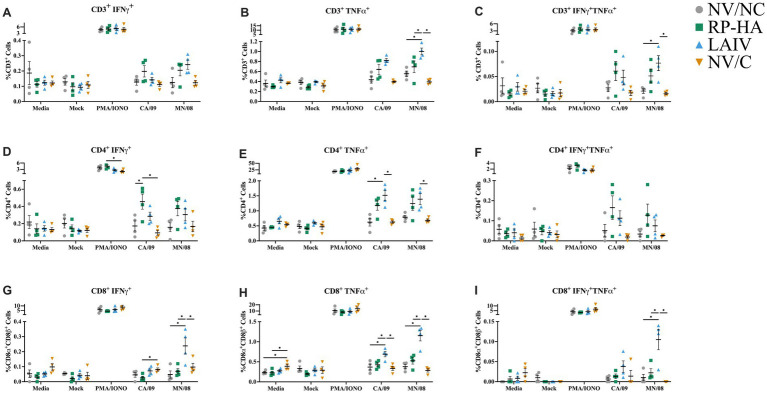
Virus-specific cytokine production of lung T cells at 5 days post-infection. Mononuclear cells isolated from lung tissue were stimulated overnight with media, mock, PMA/IONO, CA/09, or MN/08 virus (MOI 0.1). CD3^+^ **(A–C)**, CD4^+^ **(D–F)**, and CD8β ^+^ **(G–I)** production of IFN-γ **(A,D,G)**, TNF-α **(B,E,H)**, and dual IFN-γ and TNF-α **(C,F,I)**. PMA/IONO, phorbol myristate acetate and ionomycin; NV, non-vaccinated; NC, non-challenged; RP, replicon particle; HA, hemagglutinin; LAIV, live attenuated influenza virus; C, challenged; MOI, multiplicity of infection. Data are presented as mean ± standard error of the mean (*N* = 4). Statistically significant differences (*p* ≤ 0.05) between means within each stimulation treatment are indicated by lines and asterisks.

Single-cell lung suspensions were stimulated *ex vivo* with wild-type CA/09 or MN/08 to assess production of IFN-*γ* and TNF-*α* in CD3e, CD4a, and CD8β T cells (Figure 6). The gating scheme for this analysis is shown in Supplementary Figure 3. CD3^+^ cells from LAIV-vaccinated pigs had significantly increased TNF-*α* and dual IFN-γ/TNF-α production to MN/08 compared to other groups (Figures 6A–C). CD4^+^ cells from RP-HA-vaccinated pigs had significantly increased IFN-γ^+^ responses to CA/09, and though not statistically significant, RP-HA-vaccinated pigs also had elevated mean percentages of TNF-α and dual IFN-*γ*/TNF-α-producing CD4^+^ T cells in response to both CA/09 and MN/08 stimulations (Figures 6D–F). CD4^+^ cells from LAIV-vaccinated pigs had significantly increased TNF-α^+^ responses to both viral stimulations (Figure 6E). Within CD8^+^ T cells, IFN-γ^+^ and IFN-γ^+^ TNF-α^+^ cells were significantly increased in LAIV-vaccinated pigs stimulated with MN/08 compared to all other groups, and TNF-α^+^ cells were significantly increased in response to both viral stimulations (Figures 6G–I). As a control for differences in internal genes, cells from LAIV-vaccinated animals were also stimulated with the CA/09 LAIV vaccine and were similar to CA/09 stimulation (Supplementary Figure 4). Together, this data demonstrates LAIV-vaccinated pigs had higher cytokine responses to CA/09 vaccine and MN/08 challenge virus stimulation, except for elevated lung CD4^+^ IFN-γ responses in RP-HA-vaccinated pigs.

### 3.5 Experimental RP-HA and LAIV vaccines induced variable lung T cell proliferation responses

**Figure 7:**
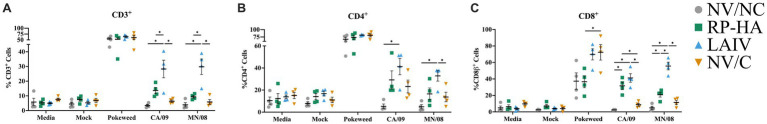
Virus-specific proliferation of lung T cells at 5 DPI. Mononuclear cells isolated from lung tissue were stained with CellTrace Violet and stimulated for 6 days with media, mock, pokeweed mitogen, CA/09, or MN/08 virus (MOI 0.1). CTV^low^ percentages of CD3^+^ **(A)**, CD4^+^ **(B)**, and CD8β^+^ **(C)** populations are shown. CTV, CellTrace Violet; NV, non-vaccinated; NC, non-challenged; RP, replicon particle; HA, hemagglutinin; LAIV, live attenuated influenza virus; C, challenged; MOI, multiplicity of infection. Data are presented as mean ± standard error of the mean (*N* = 4). Statistically significant differences (*p* ≤ 0.05) between means within each stimulation treatment are indicated by lines and asterisks.

Single-cell lung suspensions were also stained with cell trace violet and stimulated with CA/09 or MN/08 virus, and T cell subsets (CD3e, CD4a, and CD8β positive cells) were evaluated for proliferation. The gating scheme for this analysis is shown in Supplementary Figure 5. LAIV-vaccinated pigs responded to both viral stimulations with significantly increased proliferation of CD3^+^ cells compared to all other groups, and significant increases in proliferation to both viral stimulations were also noted for CD4^+^ and CD8^+^ T cell subsets of LAIV-vaccinated pigs (Figures 7A–C). While not always statistically significant, RP-HA-vaccinated pigs also had elevated mean percentages of proliferating cells in response to both viral stimulations, and significant increases in proliferation were noted for CD8^+^ T cell-stimulated with both viruses for RP-HA-vaccinated pigs relative to unvaccinated pigs (Figures 7A–C). As a control for differences in internal genes, cells from LAIV-vaccinated animals were also stimulated with the CA/09 LAIV vaccine and were similar to CA/09 stimulation (Supplementary Figure 6).

## 4 Discussion

There is a critical need for improvement of swine IAV vaccination strategies to increase the breadth of immunity beyond neutralizing antibody in order to elicit superior protection against heterologous infection. To better understand mechanisms underlying vaccine immunity, this study characterized antibody and cellular immune responses following administration of experimental LAIV and RP-HA IAV vaccines and subsequent heterologous IAV infection. LAIV vaccines have previously been shown to have superior heterologous protection compared to WIV vaccines (17), but have not been evaluated in parallel to the RP-HA vaccine platform for heterologous infection. Prior studies indicate that LAIV heterologous immunity is dependent on the hemagglutinin antigenic relationship between the vaccine and the infecting virus (15, 16, 50, 51). RP-HA vaccines have more limited research evaluation of heterologous immunity, but it is likely also dependent on the vaccine and the infection virus’s hemagglutinin antigenic relationship (7, 23).

In the present study, RP-HA-vaccinated pigs had reduced macroscopic lung lesions compared to LAIV and a trend for reduction compared to NV/C. Previous studies with this LAIV platform and RP-HA demonstrated a range in reduction in macroscopic and microscopic lesion scores with homologous and heterologous challenge, indicating that protection from heterologous viruses is likely dependent on the antigenic diversity between the two viruses (7, 15, 16, 23, 50). The virus combination of CA/09 and MN/08 has been well-documented to induce VAERD with WIV vaccines, with either combination of vaccine and challenge virus (11, 13, 15, 16, 50, 52). In the present study, neither of the vaccines induced VAERD in accordance with past studies using LAIV and RP vaccines (7, 16, 50, 53). Though swine IAV has evolved and diversified since CA/09 and MN/08 were isolated, H1N1 clade 1A.3.3.2 and H1N2 clade 1B.2.2 continue to circulate (37), providing relevance for the use of these virus isolates in heterologous vaccine/challenge studies.

Experimental swine LAIV vaccines have been demonstrated to reduce or eliminate viral shedding and transmission following heterologous infection (15, 16, 50, 51). In the present study, LAIV-vaccinated pigs had significantly reduced viral titers in nasal swabs and BALF compared to both RP-HA and NV/C. A prior study with the same LAIV platform and virus combination demonstrated minimal virus detection in nasal swabs and no detection in BALF of LAIV-vaccinated pigs (15). Two prior studies with the same LAIV platform but opposite vaccine and challenge viruses demonstrated minimal to no virus detection in LAIV vaccinated pigs, and an additional study with a different heterologous virus combination had no virus detection after challenge (14, 16, 50). In a previous evaluation of heterologous infection with RP vaccines using a different virus combination, RP-vaccinated pigs had reduced virus titers in the lung compared to NV/C but equivalent titers in nasal swabs (7). Another study with RP vaccines showed a reduction in viral titers with a heterologous vaccine and challenge virus (23).

Serum antibody to HA, measured by hemagglutinin inhibition assay, has been established as the gold standard correlate of protection for influenza vaccines. Traditional adjuvanted inactivated swine vaccines rely on the induction of high levels of systemic neutralizing antibody, which is highly effective in providing protection from homologous viral infection. In the current study, homologous serum HI titers were similar to past studies evaluating these RP and LAIV swine vaccine platforms (7, 10, 14, 15, 23, 54). The development of peripheral IgG was comparable to past studies (7, 10, 15). A previous study demonstrated NI titers induced by LAIV similar to WIV vaccines and wild-type virus (55). RP vaccines encoding NA do induce NI antibody, but this study was limited to RP-HA (6, 7).

Mucosal antibodies are present along the respiratory mucosa but are most often assessed in the lower respiratory tract using BALF, and these antibodies are important for virus binding to neutralize and block attachment at the site of infection. Several LAIV vaccine platforms have demonstrated induction of mucosal IgG and IgA that is cross-reactive to heterologous viruses, including previous studies with this same platform (14–17, 36, 53, 56). RP-HA vaccines have also been demonstrated to induce virus-specific mucosal IgG and, to a lesser extent, IgA (7, 23). In the present study, LAIV induced higher levels of mucosal antibody than RP-HA, which was cross-reactive to the challenge virus, while RP-HA primarily induced homologous IgG.

T cells have multiple roles in protection from the influenza virus, such as activation of B cells and direct killing of infected cells, and memory T cells induced by vaccination are likely integral in vaccine protection (25, 30, 31, 33, 57). In swine, multifunctional T cells producing proinflammatory cytokines (IFN-*γ* and TNF-*α*) have been demonstrated to be activated with influenza infection in the lung and periphery, and most display a memory phenotype (25, 30, 31). Experimental swine LAIV vaccines induce cellular immune responses, such as IFN-γ production and virus-specific proliferation responses (10, 15, 26–28, 36). Previous T cell data from swine RP vaccines demonstrate induction of peripheral IFN-*γ* production by PBMCs (22, 29). In the current study, both vaccines induced T cell production of proinflammatory cytokines and proliferation in response to vaccine and challenge virus stimulation at 5DPI. However, the magnitude of T cell proinflammatory cytokine production and proliferation was generally larger in LAIV-vaccinated pigs. As a live mucosal vaccine, LAIV infects epithelial cells, similar to a natural viral infection, and subsequently primes T cells, including CD8^+^ T cells (10, 32, 58, 59). Upon heterologous infection, those primed CD8^+^ cells are likely activated by internal genes or conserved HA or NA viral epitopes (58, 59). Results of our study indicate that LAIV stimulates T cell activation (i.e., proliferation and cytokine production), concordant with previous findings of T cells being a primary target for vaccine-induced adaptive memory recall with swine LAIV vaccines, and is likely a correlate of heterologous protection (10, 19, 26, 32).

RP-vaccinated pigs had a significantly increased IFN-*γ* CD4^+^ T cell responses compared to NV/C and NV/NC, and a trend for increased lung IFN-γ and dual IFN-γ and TNF-*α* CD4^+^ T cell responses compared to LAIV. RNA-based vaccines, including alphavirus-based replicon particle vaccines, are administered peripherally and likely taken up by antigen-presenting cells, and are thought to activate a less humoral-biased immune response than traditional adjuvanted, inactivated influenza virus vaccines (24, 60). Upon heterologous infection following administration of an RNA-based HA vaccine, conserved epitopes on the hemagglutinin reactivate CD4^+^ T cells that may play important roles in promoting antiviral immunity through IFN-γ production. RP-HA-vaccinated pigs also had increased lung CD8^+^ T cell proliferation, indicating mucosal activation of antiviral effector cells. These data demonstrate that peripherally administered RP-HA vaccines can elicit T cell responses at the mucosa, and further studies to fully characterize these mucosal T cells would be beneficial to determine if they are crucial tissue-resident memory T cells. Other T cell subsets, such as gamma delta T cells and lung tissue-resident memory T cells, are also likely important in vaccine responses to IAV in swine, but they were not assessed in this study, though they would be included in the overall CD3^+^ T cell populations that were assessed (32, 61).

In this study, we demonstrated that LAIV and RP-HA IAV vaccines induce differential antibody and T cell responses. Additionally, the vaccines induced differences in vaccine protection, as LAIV limited viral shedding and viral lung load while RP-HA limited macroscopic lung lesions. Though this study had small group sizes, significant differences were still noted, and with a larger sample size, more differences may have been significant. These data emphasize that immune responses to vaccines are dependent on the platform and route of exposure, and there is a need to further investigate beyond traditional WIV vaccines to improve heterologous protection. Neuraminidase has recently been approved for commercially available swine RP vaccines, and nucleoprotein has been experimentally evaluated in swine (6, 29). Due to the potential for reassortment with viruses in nature, LAIV is unlikely to be commercially available for swine unless further measures to prevent reassortment are implemented (20). Recent studies have evaluated a chimeric bat-swine reassortment-incompetent LAIV platform in swine, with internal gene segments and packaging signals from a bat IAV on the swine HA and NA gene segments (62, 63). Further evaluation of reassortment incompetent LAIV platforms and other alternative platforms will assist in improving vaccine strategies with enhanced efficacy against the diversity of circulating swine influenza A viruses. Continued assessment of mucosal antibody targets and CD4^+^ and CD8^+^ T cell epitopes is necessary to improve heterologous IAV vaccine protection. Additionally, a better understanding of heterologous correlates of protection, including enhanced CD8^+^ T cell responses contributing to decreased viral shedding, will aid in the development of improved swine IAV vaccine strategies.

## Data Availability

The datasets presented in this study can be found in online repositories. The names of the repository/repositories and accession number(s) can be found at: USDA Ag Data Commons, https://doi.org/10.15482/USDA.ADC/29591348.

## References

[1] Anderson TK Chang J Arendsee ZW Venkatesh D Souza CK Kimble JB . Swine influenza A viruses and the tangled relationship with humans. Cold Spring Harb Perspect Med. (2021) 11:a038737. doi: 10.1101/cshperspect.a038737 31988203 PMC7919397

[2] Markin A Ciacci Zanella G Arendsee ZW Zhang J Krueger KM Gauger PC . Reverse-zoonoses of 2009 H1N1 pandemic influenza A viruses and evolution in United States swine results in viruses with zoonotic potential. PLoS Pathog. (2023) 19:e1011476. doi: 10.1371/journal.ppat.1011476, 37498825 PMC10374098

[3] Vincent AL Perez DR Rajao D Anderson TK Abente EJ Walia RR . Influenza a virus vaccines for swine. Vet Microbiol. (2017) 206:35–44. doi: 10.1016/j.vetmic.2016.11.026, 27923501 PMC8609643

[4] Kaplan BS Souza CK Kimble JB Brand MW Anderson TK Gauger PC . A neuraminidase-based inactivated influenza virus vaccine significantly reduced virus replication and pathology following homologous challenge in swine. Vaccine. (2025) 46:126574. doi: 10.1016/j.vaccine.2024.126574, 39645432 PMC11874874

[5] Eichelberger MC Monto AS. Neuraminidase, the forgotten surface antigen, emerges as an influenza vaccine target for broadened protection. J Infect Dis. (2019) 219:S75–80. doi: 10.1093/infdis/jiz017, 30715357 PMC7325326

[6] Kitikoon P Knetter SM Mogler MA Morgan CL Hoehn A Puttamreddy S . Quadrivalent neuraminidase RNA particle vaccine protects pigs against homologous and heterologous strains of swine influenza virus infection. Vaccine. (2023) 41:6941–51. doi: 10.1016/j.vaccine.2023.10.005 37884412

[7] Wymore Brand M Anderson TK Kitikoon P Brian Kimble J Otis N Gauger PC . Bivalent hemagglutinin and neuraminidase influenza replicon particle vaccines protect pigs against influenza A virus without causing vaccine associated enhanced respiratory disease. Vaccine. (2022) 40:5569–78. doi: 10.1016/j.vaccine.2022.07.042, 35987871

[8] Petro-Turnquist E Pekarek MJ Weaver EA. Swine influenza A virus: challenges and novel vaccine strategies. Front Cell Infect Microbiol. (2024) 14:1336013. doi: 10.3389/fcimb.2024.1336013, 38633745 PMC11021629

[9] Sandbulte MR Spickler AR Zaabel PK Roth JA. Optimal use of vaccines for control of influenza A virus in swine. Vaccine. (2015) 3:22–73. doi: 10.3390/vaccines3010022, 26344946 PMC4494241

[10] Loving CL Vincent AL Pena L Perez DR. Heightened adaptive immune responses following vaccination with a temperature-sensitive, live-attenuated influenza virus compared to adjuvanted, whole-inactivated virus in pigs. Vaccine. (2012) 30:5830–8. doi: 10.1016/j.vaccine.2012.07.033, 22835742 PMC3743435

[11] Gauger PC Vincent AL Loving CL Henningson JN Lager KM Janke BH . Kinetics of lung lesion development and pro-inflammatory cytokine response in pigs with vaccine-associated enhanced respiratory disease induced by challenge with pandemic (2009) a/H1N1 influenza virus. Vet Pathol. (2012) 49:900–12. doi: 10.1177/0300985812439724 22461226

[12] Vincent AL Lager KM Janke BH Gramer MR Richt JA. Failure of protection and enhanced pneumonia with a US H1N2 swine influenza virus in pigs vaccinated with an inactivated classical swine H1N1 vaccine. Vet Microbiol. (2008) 126:310–23. doi: 10.1016/j.vetmic.2007.07.011, 17719188

[13] Souza CK Rajao DS Sandbulte MR Lopes S Lewis NS Loving CL . The type of adjuvant in whole inactivated influenza A virus vaccines impacts vaccine-associated enhanced respiratory disease. Vaccine. (2018) 36:6103–10. doi: 10.1016/j.vaccine.2018.08.072, 30181048

[14] Pena L Vincent AL Ye J Ciacci-Zanella JR Angel M Lorusso A . Modifications in the polymerase genes of a swine-like triple-reassortant influenza virus to generate live attenuated vaccines against 2009 pandemic H1N1 viruses. J Virol. (2011) 85:456–69. doi: 10.1128/JVI.01503-10, 20962084 PMC3014183

[15] Rajao DS Loving CL Gauger PC Kitikoon P Vincent AL. Influenza a virus hemagglutinin protein subunit vaccine elicits vaccine-associated enhanced respiratory disease in pigs. Vaccine. (2014) 32:5170–6. doi: 10.1016/j.vaccine.2014.07.059, 25077416

[16] Gauger PC Loving CL Khurana S Lorusso A Perez DR Kehrli ME Jr . Live attenuated influenza A virus vaccine protects against A(H1N1)pdm09 heterologous challenge without vaccine associated enhanced respiratory disease. Virology. (2014) 471–473:93–104. doi: 10.1016/j.virol.2014.10.003 25461535

[17] Abente EJ Rajao DS Santos J Kaplan BS Nicholson TL Brockmeier SL . Comparison of adjuvanted-whole inactivated virus and live-attenuated virus vaccines against challenge with contemporary, antigenically distinct H3N2 influenza A viruses. J Virol. (2018) 92:e01323-18. doi: 10.1128/JVI.01323-18 PMC6206469 30185589

[18] Richt JA Lekcharoensuk P Lager KM Vincent AL Loiacono CM Janke BH . Vaccination of pigs against swine influenza viruses by using an NS1-truncated modified live-virus vaccine. J Virol. (2006) 80:11009–18. doi: 10.1128/jvi.00787-06, 16943300 PMC1642165

[19] Masic A Lu X Li J Mutwiri GK Babiuk LA Brown EG . Immunogenicity and protective efficacy of an elastase-dependent live attenuated swine influenza virus vaccine administered intranasally in pigs. Vaccine. (2010) 28:7098–108. doi: 10.1016/j.vaccine.2010.08.003, 20708697

[20] Sharma A Zeller MA Li G Harmon KM Zhang J Hoang H . Detection of live attenuated influenza vaccine virus and evidence of reassortment in the U.S. swine population. J Vet Diagn Invest. (2020) 32:301–11. doi: 10.1177/1040638720907918, 32100644 PMC7081507

[21] Bosworth B Erdman MM Stine DL Harris I Irwin C Jens M . Replicon particle vaccine protects swine against influenza. Comp Immunol Microbiol Infect Dis. (2010) 33:e99–e103. doi: 10.1016/j.cimid.2010.05.002, 21094422

[22] Vander Veen RL Loynachan AT Mogler MA Russell BJ Harris DL Kamrud KI. Safety, immunogenicity, and efficacy of an alphavirus replicon-based swine influenza virus hemagglutinin vaccine. Vaccine. (2012) 30:1944–50. doi: 10.1016/j.vaccine.2012.01.030, 22269873

[23] Abente EJ Rajao DS Gauger PC Vincent AL. Alphavirus-vectored hemagglutinin subunit vaccine provides partial protection against heterologous challenge in pigs. Vaccine. (2019) 37:1533–9. doi: 10.1016/j.vaccine.2018.12.071, 30723064 PMC6990457

[24] Vander Veen RL Harris DL Kamrud KI. Alphavirus replicon vaccines. Anim Health Res Rev. (2012) 13:1–9. doi: 10.1017/S1466252312000011, 22436454

[25] Martini V Edmans M Gubbins S Jayaraman S Paudyal B Morgan S . Spatial, temporal and molecular dynamics of swine influenza virus-specific CD8 tissue resident memory T cells. Mucosal Immunol. (2022) 15:428–42. doi: 10.1038/s41385-021-00478-4, 35145208 PMC9038527

[26] Kappes MA Sandbulte MR Platt R Wang C Lager KM Henningson JN . Vaccination with NS1-truncated H3N2 swine influenza virus primes T cells and confers cross-protection against an H1N1 heterosubtypic challenge in pigs. Vaccine. (2012) 30:280–8. doi: 10.1016/j.vaccine.2011.10.098, 22067263

[27] Olson ZF Sandbulte MR Souza CK Perez DR Vincent AL Loving CL. Factors affecting induction of peripheral IFN-gamma recall response to influenza A virus vaccination in pigs. Vet Immunol Immunopathol. (2017) 185:57–65. doi: 10.1016/j.vetimm.2017.01.009, 28242003

[28] Masic A Booth JS Mutwiri GK Babiuk LA Zhou Y. Elastase-dependent live attenuated swine influenza A viruses are immunogenic and confer protection against swine influenza A virus infection in pigs. J Virol. (2009) 83:10198–210. doi: 10.1128/jvi.00926-09, 19625412 PMC2748057

[29] Vander Veen RL Mogler MA Russell BJ Loynachan AT Harris DL Kamrud KI. Haemagglutinin and nucleoprotein replicon particle vaccination of swine protects against the pandemic H1N1 2009 virus. Vet Rec. (2013) 173:344. doi: 10.1136/vr.101741, 24078226

[30] Talker SC Koinig HC Stadler M Graage R Klingler E Ladinig A . magnitude and kinetics of multifunctional CD4+ and CD8β+ T cells in pigs infected with swine influenza A virus. Vet Res. (2015) 46:52. doi: 10.1186/s13567-015-0182-3, 25971313 PMC4429459

[31] Talker SC Stadler M Koinig HC Mair KH Rodriguez-Gomez IM Graage R . Influenza a virus infection in pigs attracts multifunctional and cross-reactive T cells to the lung. J Virol. (2016) 90:9364–82. doi: 10.1128/jvi.01211-16, 27512056 PMC5044846

[32] Holzer B Martini V Edmans M Tchilian E. T and B cell immune responses to influenza viruses in pigs. Front Immunol. (2019) 10:98. doi: 10.3389/fimmu.2019.00098, 30804933 PMC6371849

[33] Muir A Paudyal B Schmidt S Sedaghat-Rostami E Chakravarti S Villanueva-Hernandez S . Single-cell analysis reveals lasting immunological consequences of influenza infection and respiratory immunization in the pig lung. PLoS Pathog. (2024) 20:e1011910. doi: 10.1371/journal.ppat.1011910, 39024231 PMC11257366

[34] Souquette A Thomas PG. Past life and future effects-how heterologous infections alter immunity to influenza viruses. Front Immunol. (2018) 9:1071. doi: 10.3389/fimmu.2018.01071, 29872429 PMC5972221

[35] Tchilian E Holzer B. Harnessing local immunity for an effective universal swine influenza vaccine. Viruses. (2017) 9:98. doi: 10.3390/v9050098, 28475122 PMC5454411

[36] Sandbulte MR Platt R Roth JA Henningson JN Gibson KA Rajao DS . Divergent immune responses and disease outcomes in piglets immunized with inactivated and attenuated H3N2 swine influenza vaccines in the presence of maternally-derived antibodies. Virology. (2014) 464:45–54. doi: 10.1016/j.virol.2014.06.027 25043588

[37] Janzen GM Inderski BT Chang J Arendsee ZW Janas-Martindale A Torchetti MK . Sources and sinks of influenza A virus genomic diversity in swine from 2009 to 2022 in the United States. J Virol. (2025) 99:e0054125. doi: 10.1128/jvi.00541-25, 40985717 PMC12455956

[38] Center for Veterinary Biologics. Notice NO. 17-01. 2017.

[39] Classe HM Dant JC Mogler M Stachura KA LaFleur RL Xu Z . Efficacy and safety in dogs following administration of an alphavirus RNA particle canine influenza H3N2 vaccine. Vaccine. (2024) 12:1138. doi: 10.3390/vaccines12101138, 39460305 PMC11511248

[40] Carritt K Davis R Stachura K Crumley P Mogler M Stahl M . A novel, safe, non-adjuvanted alphavirus replicon-based vaccine expressing the feline leukemia virus envelope protein protects against virulent FeLV challenge. Vaccine. (2025) 13:697. doi: 10.3390/vaccines13070697, 40733674 PMC12298305

[41] Lager KM Vincent AL. In vivo models for pathotyping and vaccine efficacy for swine influenza. Methods Mol Biol. (2020) 2123:345–51. doi: 10.1007/978-1-0716-0346-8_25, 32170700

[42] Halbur PG Paul PS Frey ML Landgraf J Eernisse K Meng XJ . Comparison of the pathogenicity of two US porcine reproductive and respiratory syndrome virus isolates with that of the Lelystad virus. Vet Pathol. (1995) 32:648–60. doi: 10.1177/030098589503200606, 8592800

[43] Morgan SB Hemmink JD Porter E Harley R Shelton H Aramouni M . Aerosol delivery of a candidate universal influenza vaccine reduces viral load in pigs challenged with pandemic H1N1 virus. J Immunol. (2016) 196:5014–23. doi: 10.4049/jimmunol.1502632, 27183611 PMC4891568

[44] Kitikoon P Nilubol D Erickson BJ Janke BH Hoover TC Sornsen SA . The immune response and maternal antibody interference to a heterologous H1N1 swine influenza virus infection following vaccination. Vet Immunol Immunopathol. (2006) 112:117–28. doi: 10.1016/j.vetimm.2006.02.008 16621020

[45] Reed LJ Muench H. A simple method of estimating fifty percent endpoints. Am J Hyg. (1938) 27:493–7. doi: 10.1093/oxfordjournals.aje.a118408

[46] Kitikoon P Gauger PC Vincent AL. Hemagglutinin inhibition assay with swine sera. Methods Mol Biol. (2014) 1161:295–301. doi: 10.1007/978-1-4939-0758-8_24, 24899438

[47] Kaplan BS Vincent AL. Detection and titration of influenza A virus neuraminidase inhibiting (NAI) antibodies using an enzyme-linked lectin assay (ELLA). Methods Mol Biol. (2020) 2123:335–44. doi: 10.1007/978-1-0716-0346-8_24, 32170699

[48] Kaplan BS Anderson TK Chang J Santos J Perez D Lewis N . Evolution and antigenic advancement of N2 neuraminidase of swine influenza A viruses circulating in the United States following two separate introductions from human seasonal viruses. J Virol. (2021) 95:e0063221. doi: 10.1128/jvi.00632-21, 34379513 PMC8475526

[49] Gauger PC Vincent AL. Enzyme-linked immunosorbent assay for detection of serum or mucosal isotype-specific IgG and IgA whole-virus antibody to influenza A virus in swine. Methods Mol Biol. (2020) 2123:311–20. doi: 10.1007/978-1-0716-0346-8_22, 32170697

[50] Souza CK Rajão DS Loving CL Gauger PC Pérez DR Vincent AL. Age at vaccination and timing of infection do not Alter vaccine-associated enhanced respiratory disease in influenza A virus-infected pigs. Clin Vaccine Immunol. (2016) 23:470–82. doi: 10.1128/CVI.00563-15, 27030585 PMC4895012

[51] Loving CL Lager KM Vincent AL Brockmeier SL Gauger PC Anderson TK . Efficacy in pigs of inactivated and live attenuated influenza virus vaccines against infection and transmission of an emerging H3N2 similar to the 2011-2012 H3N2v. J Virol. (2013) 87:9895–903. doi: 10.1128/JVI.01038-13, 23824815 PMC3754103

[52] Rajao DS Chen H Perez DR Sandbulte MR Gauger PC Loving CL . Vaccine-associated enhanced respiratory disease is influenced by haemagglutinin and neuraminidase in whole inactivated influenza virus vaccines. J Gen Virol. (2016) 97:1489–99. doi: 10.1099/jgv.0.000468, 27031847

[53] Vincent AL Ma W Lager KM Richt JA Janke BH Sandbulte MR . Live attenuated influenza vaccine provides superior protection from heterologous infection in pigs with maternal antibodies without inducing vaccine-associated enhanced respiratory disease. J Virol. (2012) 86:10597–605. doi: 10.1128/jvi.01439-12, 22811541 PMC3457301

[54] Erdman MM Kamrud KI Harris DL Smith J. Alphavirus replicon particle vaccines developed for use in humans induce high levels of antibodies to influenza virus hemagglutinin in swine: proof of concept. Vaccine. (2010) 28:594–6. doi: 10.1016/j.vaccine.2009.10.015, 19853679

[55] Sandbulte MR Gauger PC Kitikoon P Chen H Perez DR Roth JA . Neuraminidase inhibiting antibody responses in pigs differ between influenza A virus N2 lineages and by vaccine type. Vaccine. (2016) 34:3773–9. doi: 10.1016/j.vaccine.2016.06.001, 27325350

[56] Rajao DS Zanella GC Wymore Brand M Khan S Miller ME Ferreri LM . Live attenuated influenza A virus vaccine expressing an IgA-inducing protein protects pigs against replication and transmission. Front Virol. (2023) 3:1042724. doi: 10.3389/fviro.2023.1042724

[57] Sant AJ DiPiazza AT Nayak JL Rattan A Richards KA. CD4 T cells in protection from influenza virus: viral antigen specificity and functional potential. Immunol Rev. (2018) 284:91–105. doi: 10.1111/imr.12662, 29944766 PMC6070306

[58] Mohn KG Smith I Sjursen H Cox RJ. Immune responses after live attenuated influenza vaccination. Hum Vaccin Immunother. (2018) 14:571–8. doi: 10.1080/21645515.2017.1377376, 28933664 PMC5861782

[59] Shannon I White CL Nayak JL. Understanding immunity in children vaccinated with live attenuated influenza vaccine. J Pediatric Infect Dis Soc. (2020) 9:S10–4. doi: 10.1093/jpids/piz083 31848606

[60] Chaudhary N Weissman D Whitehead KA. mRNA vaccines for infectious diseases: principles, delivery and clinical translation. Nat Rev Drug Discov. (2021) 20:817–38. doi: 10.1038/s41573-021-00283-5, 34433919 PMC8386155

[61] Gerner W Mair KH Schmidt S. Local and systemic T cell immunity in fighting pig viral and bacterial infections. Annu Rev Anim Biosci. (2022) 10:349–72. doi: 10.1146/annurev-animal-013120-044226, 34724393

[62] Graaf-Rau A Schmies K Breithaupt A Ciminski K Zimmer G Summerfield A . Reassortment incompetent live attenuated and replicon influenza vaccines provide improved protection against influenza in piglets. NPJ Vaccines. (2024) 9:127. doi: 10.1038/s41541-024-00916-x, 39003272 PMC11246437

[63] Graaf A Petric PP Sehl-Ewert J Henritzi D Breithaupt A King J . Cold-passaged isolates and bat-swine influenza A chimeric viruses as modified live-attenuated vaccines against influenza A viruses in pigs. Vaccine. (2022) 40:6255–70. doi: 10.1016/j.vaccine.2022.09.013, 36137904

