# Supplementary material for: Swine influenza A replicon particle and live attenuated influenza virus vaccines induce differential systemic and mucosal antibody and T cell responses
Source: Front Vet Sci. 2026 Jan 30;12:1690418. doi: 10.3389/fvets.2025.1690418 (PMC12903121; doi:10.3389/fvets.2025.1690418)
Supplement: Supplementary file 1 [file Data_Sheet_1.docx]

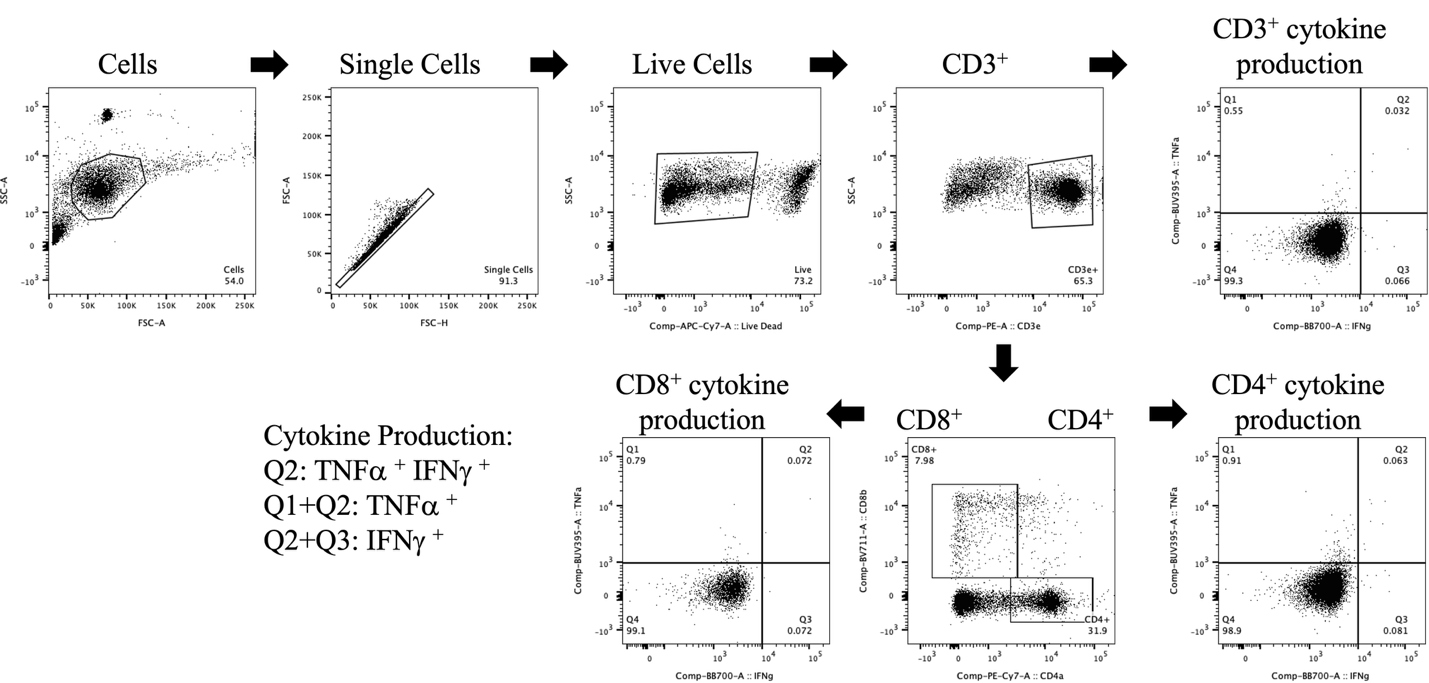


**Supplementary Figure 1.** Gating hierarchy for PBMC cytokine production. Shown are cells from an LAIV-vaccinated pig stimulated with MN/08.


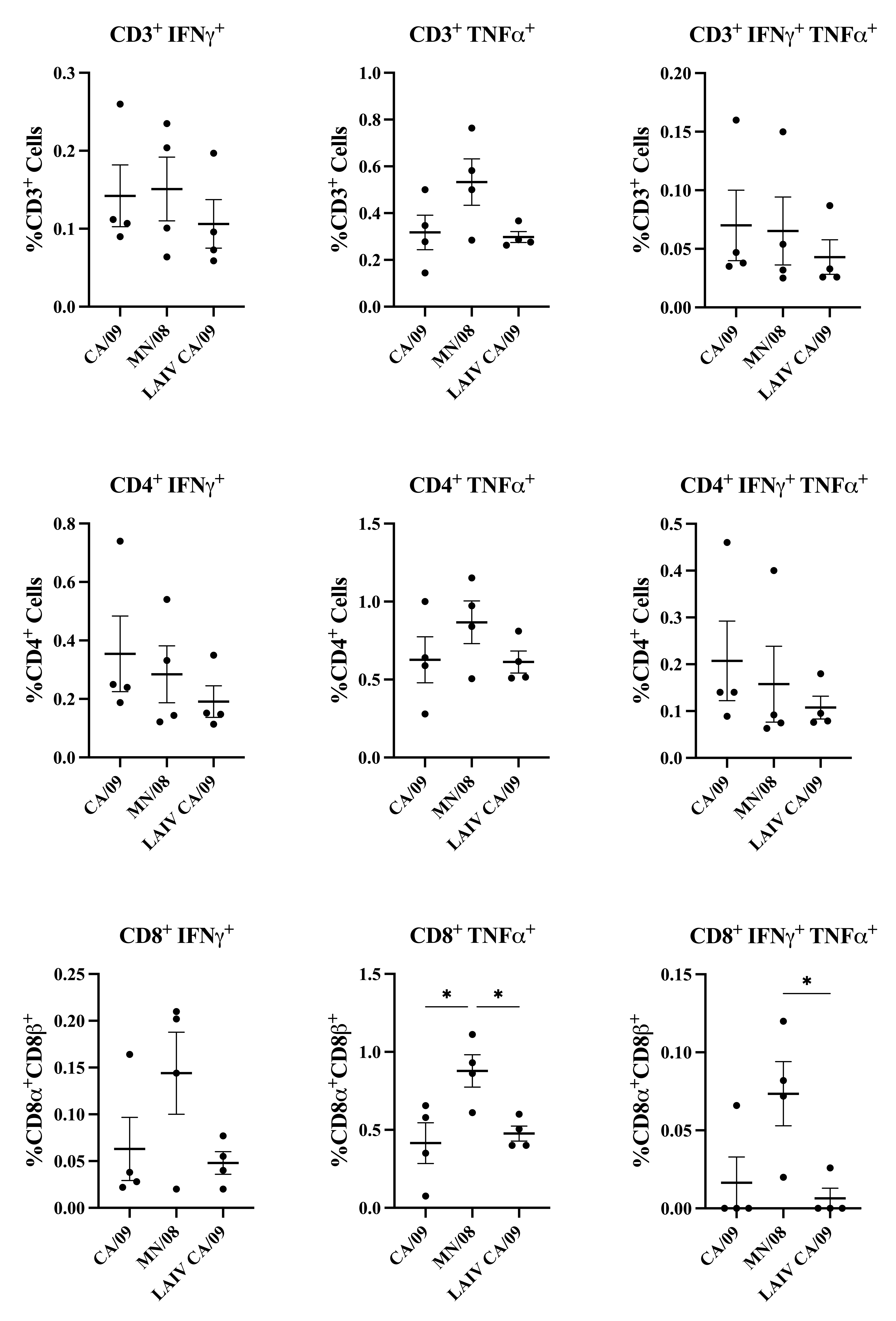


**Supplementary Figure 2.** Virus-specific cytokine production of peripheral T cells from LAIV-vaccinated animals at 5 days post-infection stimulated with CA/09, MN/08, and LAIV CA/09. Peripheral blood mononuclear cells were stimulated overnight with CA/09, MN/08, and LAIV CA/09 (MOI 0.1). CD3^+^, CD4^+^, and CD8^+^ production of IFN-γ, TNF-α, and dual IFN-γ and TNF-α. LAIV, live attenuated influenza virus; MOI, multiplicity of infection. Data presented as mean ± standard error of the mean (N=4). Statistically significant differences (*p* ≤ 0.05) between means within each stimulation treatment are indicated by lines and asterisks.


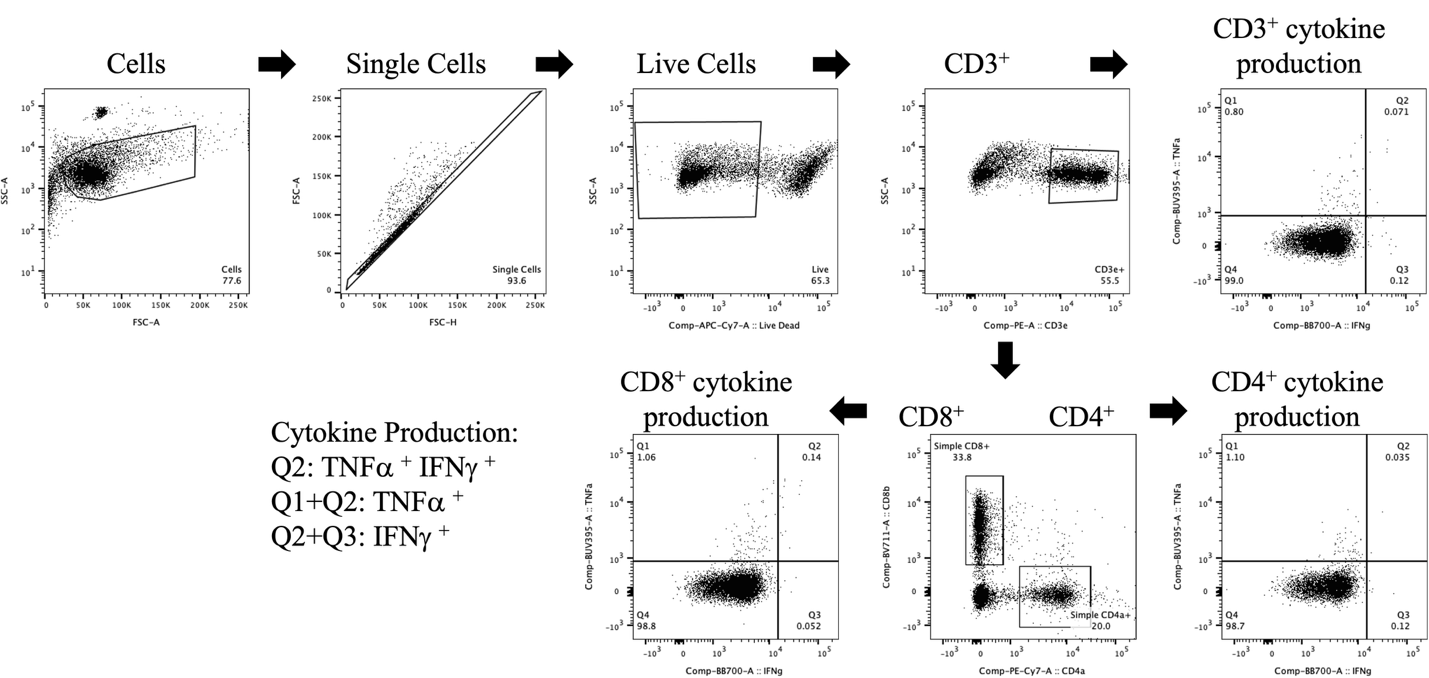


**Supplementary Figure 3.** Gating hierarchy for lung mononuclear cell cytokine production. Shown are cells from an LAIV-vaccinated pig stimulated with MN/08.


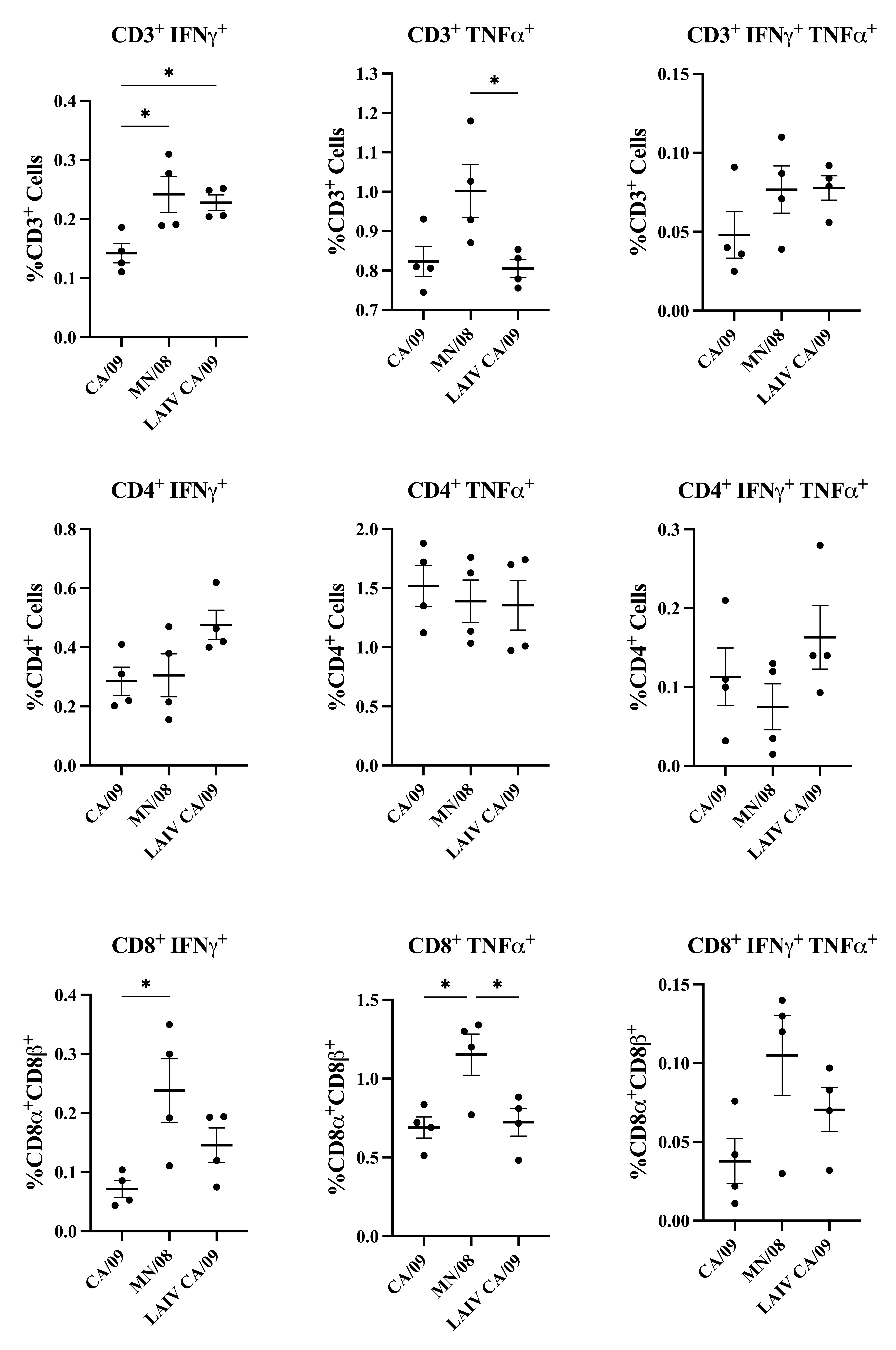


**Supplementary Figure 4.** Virus-specific cytokine production of lung T cells from LAIV-vaccinated animals at 5 days post-infection stimulated with CA/09, MN/08, and LAIV CA/09. Peripheral blood mononuclear cells were stimulated overnight with CA/09, MN/08, and LAIV CA/09 (MOI 0.1). CD3^+^, CD4^+^, and CD8^+^ production of IFN-γ, TNF-α, and dual IFN-γ and TNF-α. LAIV, live attenuated influenza virus; MOI, multiplicity of infection. Data presented as mean ± standard error of the mean (N=4). Statistically significant differences (*p* ≤ 0.05) between means within each stimulation treatment are indicated by lines and asterisks.


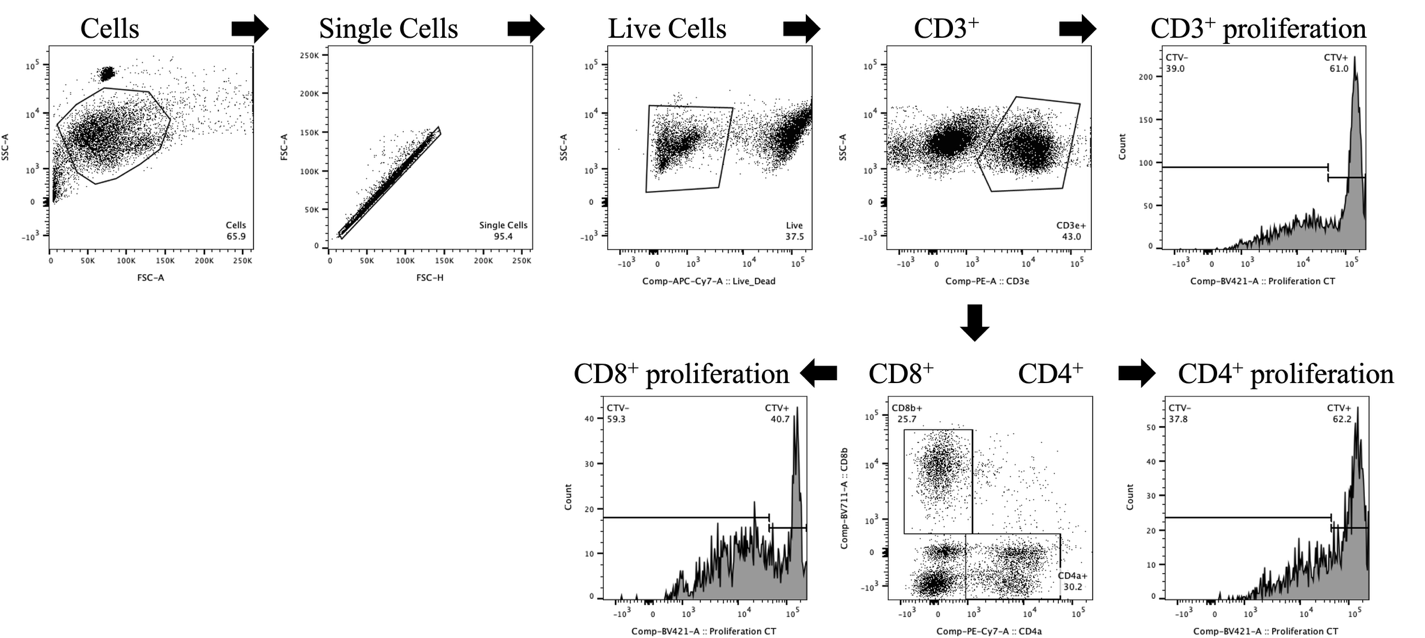


**Supplementary Figure 5.** Gating hierarchy for lung mononuclear cell proliferation. Shown are cells from an LAIV-vaccinated pig stimulated with MN/08.


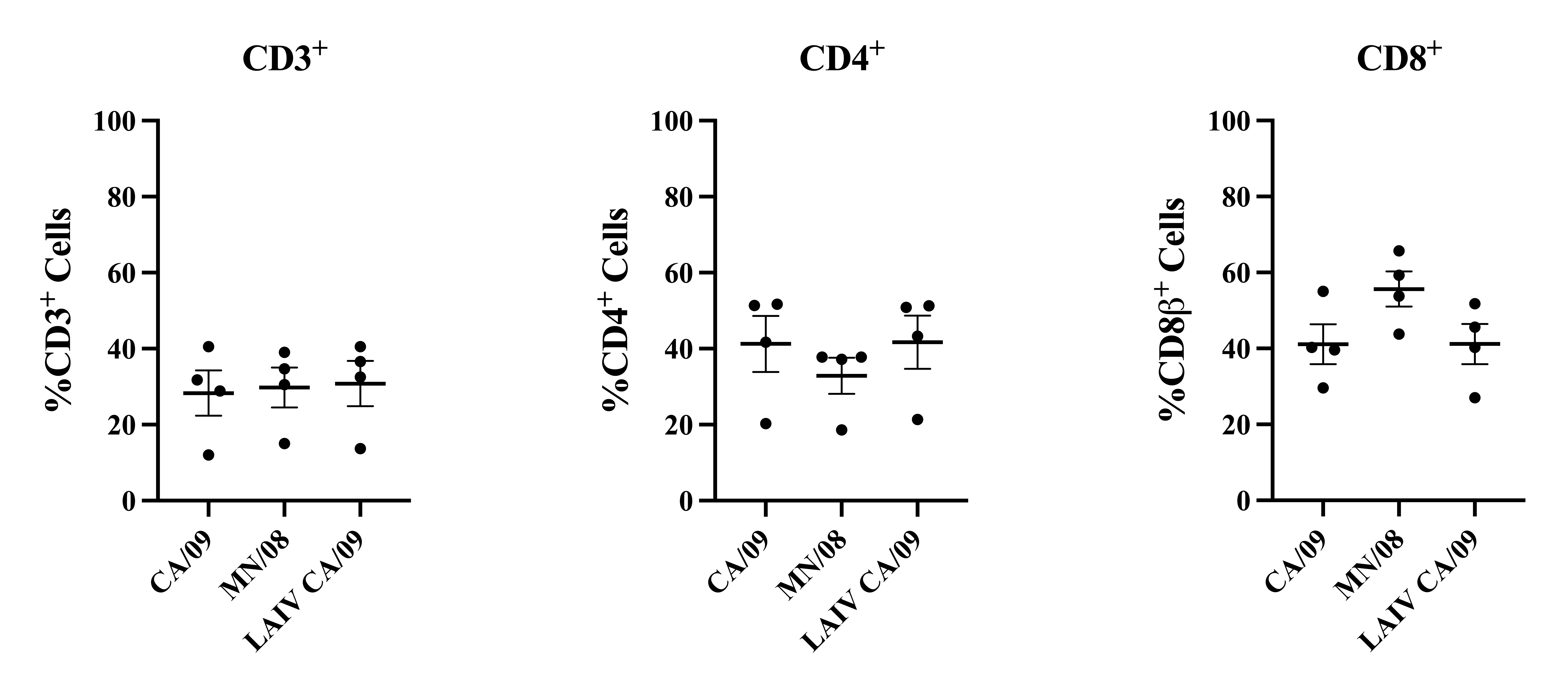


**Supplementary Figure 6.** Virus-specific proliferation of lung T cells at 5DPI stimulated with CA/09, MN/08, and LAIV CA/09. Mononuclear cells isolated from lung tissue were stained with CellTrace^TM^ Violet and stimulated for 6 days with with CA/09, MN/08, and LAIV CA/09 (MOI 0.1). CTV^low^ percentages of CD3^+^, CD4^+^, and CD8^+^ populations are shown. CTV, CellTrace Violet; LAIV, live attenuated influenza virus; MOI, multiplicity of infection. Data presented as mean ± standard error of the mean (N=4). Statistically significant differences (*p* ≤ 0.05) between means within each stimulation treatment are indicated by lines and asterisks.

**Supplementary Table 1.** Swine macroscopic lesion scale for weighted average calculation

| **Lung Lobe** | **Weighted Proportion of Total Lung** |
| --- | --- |
| Right Cranial | 10 % |
| Right Middle | 10 % |
| Right Accessory | 5 % |
| Right Caudal | 27.5 % |
| Cranial Part of Left Cranial | 10 % |
| Caudal Part of Left Cranial | 10 % |
| Left Caudal | 27.5 % |
| **SUM** | **100 %** |

Adapted from (1)

**Supplementary Table 2.** Swine microscopic lung lesion scoring system

|  | **0** | **1** | **2** | **3** | **4** |
| --- | --- | --- | --- | --- | --- |
| Necrotizing bronchitis/bronchiolitis | None | Rare foci affecting 1-2 airways | Affecting 2 airways up to 33% | Affecting 34%-66% of airways | Affecting >66% of airways |
| Suppurative bronchitis/bronchiolitis | None | Sparsely scattered granulocytic inflammation affecting occasional airways | Affecting up to 33% of airways | Affecting 34%-66% of airways | Affecting >66% of airways |
| Peribronchiolar/vascular lymphocytic cuffing | None | Occasional incomplete, loosely formed | Numerous, predominantly incomplete and loosely formed | Numerous cuffs, half or more well-formed | Numerous cuffs, predominately well-formed |
| Alveolar septal inflammation | None | Scattered granulocytes likely in vessels | Focal or multifocal, 3-4 nuclei wide | Focal or multifocal, 5 or more nuclei wide | Coalescing to diffuse, 3 or more cell wide |
| Alveolar cellular exudate/edema/hemorrhage | None | Occasional alveoli with eosinophilic fluid | Confluent alveoli and/or septa up to 33% | Affecting 34%-66% of alveoli and/or septa | Affecting >66% of alveoli and/or septa |

Adapted from (2)

**Supplementary Table 3.** Swine microscopic tracheal lesion scoring system

|  | **0** | **1** | **2** | **3** | **4** |
| --- | --- | --- | --- | --- | --- |
| Tracheal Epithelium | None | Focal to multifocal loss of cilia with epithelial cell degenerative changes | Loss of cilia and decreased goblet cells; epithelium 2-3 cell layers thick; segmented or multifocal | Loss of cilia and decreased goblet cells; epithelium 1-2 cell layers thick; segmented or multifocal | Loss of cilia and decreased goblet cells; Single layer of cuboidal epithelium or ulceration |
| Tracheitis | None | Sparse leukocytes that are predominately intraepithelial and perivascular | Leukocytes form multifocal aggregates | Leukocytes are present circumferentially | Leukocytes are present circumferentially and extend to submucosal glands |

Adapted from (3)

References

1. Halbur PG, Paul PS, Frey ML, Landgraf J, Eernisse K, Meng XJ, et al. Comparison of the pathogenicity of two US porcine reproductive and respiratory syndrome virus isolates with that of the Lelystad virus. Vet Pathol. 1995;32(6):648-60.

2. Morgan SB, Hemmink JD, Porter E, Harley R, Shelton H, Aramouni M, et al. Aerosol Delivery of a Candidate Universal Influenza Vaccine Reduces Viral Load in Pigs Challenged with Pandemic H1N1 Virus. J Immunol. 2016;196(12):5014-23.

3. Gauger PC, Loving CL, Khurana S, Lorusso A, Perez DR, Kehrli ME, Jr., et al. Live attenuated influenza A virus vaccine protects against A(H1N1)pdm09 heterologous challenge without vaccine associated enhanced respiratory disease. Virology. 2014;471-473:93-104.
